# A scoring system to predict HBsAg seroclearance in hepatitis B and C coinfected patients treated with interferon and ribavirin in an Asian cohort

**DOI:** 10.1097/MD.0000000000013383

**Published:** 2018-12-14

**Authors:** Yi-Hao Yen, Kwong-Ming Kee, Fang-Ying Kuo, Kuo-Chin Chang, Tsung-Hui Hu, Sheng-Nan Lu, Jing-Houng Wang, Chao-Hung Hung, Chien-Hung Chen

**Affiliations:** a Division of Hepatogastroenterology, Department of Internal Medicine, Kaohsiung Chang Gung Memorial Hospital and Chang Gung University College of Medicine; b Department of Pathology, Kaohsiung Chang Gung Memorial Hospital and Chang Gung University College of Medicine, Kaohsiung, Taiwan.

**Keywords:** coinfection, HBsAg seroclearance, hepatitis B virus, hepatitis C virus, interferon

## Abstract

Current guidelines recommend that patients with hepatitis B virus-hepatitis C virus (HBV-HCV) coinfection be treated with direct anti-viral agents (DAAs). Compared with DAAs, pegylated interferon (PEG-IFN) and ribavirin therapy has the advantages of treating both viruses while maintaining an acceptable HCV sustained virological response (SVR) rate (70–80%) in Asian cohorts. In this study, we aimed to develop a simple scoring system to predict hepatitis B surface antigen (HBsAg) seroclearance in these patients. We enrolled 201 patients with HCV-HBV coinfection after IFN and ribavirin therapy. The study population was randomly allocated into derivation and validation sets in a 1:1 ratio. In the derivation cohort, multivariate analysis by Cox regression analysis revealed that HBsAg seroclearance was associated with age > 60 years (HR: 5.55, 95% CI: 1.68–18.37, *P* = .005), male gender (HR: 3.88, 95% CI: 1.18–12.80, *P* = .03), and qHBsAg level ≤100 IU/ml (HR: 4.87, 95% CI: 1.20–19.74, *P* = .03). Regression coefficients were used to build up a risk score, and the accuracy of the risk score was evaluated by using the area under the receiver operating characteristic curve (AUROC). The patients were classified into either a low-risk group or high-risk group based on the risk scores. Twenty-three (23.0%) patients in the derivation cohort and 30 (29.7%) patients in the validation cohort showed HBsAg seroclearance with an AUROC of 71.8%, sensitivity of 65.22%, and specificity of 75.32%. In the validation cohort, the 5-year HBsAg seroclearance incidence rates were 23.4% in the low-risk category and 43.8% in the high-risk category (HR = 2.21; 95% CI, 1.04–4.68, *P* = .04)

The risk scoring system could be used to predict HBsAg seroclearance for HCV-HBV coinfected patients treated with IFN and ribavirin.

## 1 Introduction

In areas with a high prevalence of hepatitis B virus (HBV) infection, such as Asia-Pacific countries including Taiwan, patients may have acquired HBV infection at birth, with hepatitis C virus (HCV) infection occurring later as a superinfection, while most patients from the USA and Europe present with HBV superinfection following chronic hepatitis C (CHC) infection.^[1]^

In Taiwan, seroprevalence studies have shown that concurrent HCV infection occurs in approximately 10% to 15% of patients with chronic HBV infection, although the prevalence rates may vary among different regions and countries.^[2–4]^ Those coinfected patients have been reported to face a significantly higher risk of developing advanced liver disease and hepatocellular carcinoma (HCC) than those with either infection alone.^[5–8]^

The European Association for the Study of the Liver (EASL) guidelines recommend that patients with HBV-HCV coinfection should be treated with the same anti-HCV regimens (ie, direct anti-viral agents, DAAs) as HCV monoinfected patients.^[9]^ A previous multicenter study in Taiwan reported that the HCV sustained virological response (SVR) rate in HBV-HCV coinfection was 72.2% for patients with HCV genotype 1 infection and 82.8% for patients with HCV genotype 2 infection after pegylated interferon (PEG-IFN) and ribavirin (RBV) therapy.^[10]^ The high HCV SVR rate in Taiwanese cohorts is reportedly due to favorable interleukin 28B genotypes.^[11]^

Prior studies have indicated that around 30% of coinfected patients lose hepatitis B surface antigen (HBsAg) within 5 years after completing IFN-based therapy.^[12–15]^ The benefit of IFN-based therapy in coinfected patients was further confirmed in a large population-based study in Taiwan. Compared to coinfected patients not treated with IFN-based therapy, the risks of developing HCC, liver-related mortality, and all-cause mortality decreased by 35%, 59%, and 62%, respectively, in coinfected patients who received IFN-based therapy.^[16]^

Therefore, IFN and RBV therapy still plays a role for HBV-HCV co-infection in Asian patients. Factors related to HBsAg seroclearance in HBV-HCV coinfected patients who receive IFN and RBV therapy have been reported.^[12–15]^ The aim of this study was to develop a simple scoring system to predict HBsAg seroclearance in these patients.

## 2 Patients and methods

### 2.1 Patient selection

This retrospective study collected data from 201 treatment-naïve chronic HCV-HBV coinfected patients with active hepatitis C who received IFN and RBV therapy during the period from 1999 to 2015 and were subsequently followed up for more than 24 weeks after the treatment in Kaoshiung Chang Gung Memorial Hospital. Twenty-eight patients received 3 or 5 million units of IFN-alpha-2b thrice weekly and RBV daily for a fixed duration of 24 weeks, while 173 patients received response-guided therapy with PEG-IFN and RBV, irrespective of their genotype. The details of the response-guided therapy were as follows: 24 weeks of PEG-IFN and RBV treatment for patients with undetectable HCV RNA at week 4; 48 weeks of treatment for patients with detectable HCV RNA at week 4 and undetectable HCV RNA at week 12; and the cessation of treatment at week 16 for patients with detectable HCV RNA at week 12. Sustained virological response (SVR) was defined as undetectable HCV RNA at follow-up week 24.^[17]^

Serum HBsAg levels were measured annually. Ultrasound and serum alpha-fetoprotein measurements for HCC surveillance were conducted every 6 months during the follow-up period for patients without liver cirrhosis. HCC surveillance was conducted every 3 months for patients with liver cirrhosis.^[18]^

Liver cirrhosis was diagnosed by histology in those who underwent liver biopsy, and 93 patients in this study underwent liver biopsy. For those who did not undergo liver biopsy, liver cirrhosis was diagnosed by ultrasound, computerized tomography scan, or magnetic resonance imaging showing cirrhosis combined with portosystemic collateral vessels, or by endoscopy showing esophageal or gastric varices.^[19]^

The inclusion criterion for the study participants was a diagnosis of HCV-HBV coinfection with active hepatitis C, which was defined by seropositivity for both anti-HCV and HBsAg for more than 6 months together with detectable serum HCV-RNA (> 50 IU/mL). Patients coinfected with HIV or diagnosed with HCC prior to the initiation of therapy were excluded. For each patient, the date of inclusion was the date of starting treatment. The final follow-up date in this study was 31 March, 2017.

All the procedures followed were in accordance with the ethical standards of the responsible committees on human experimentation (institutional and national) and with the Helsinki Declaration of 1975, as revised in 2008. This study was approved by the Institutional Review Board of Kaohsiung Chang Gung Memorial Hospital (IRB no. 105–1455C). The requirement for informed consent was waived by the IRB. The data were analyzed anonymously.

### 2.2 Quantification of HBsAg and HBV DNA

HBsAg was quantified by using a chemiluminescent microparticle immunoassay (Architect HBsAg, Abbott Diagnostics, Princeton, NJ, USA). Serum HBsAg < 0.05 IU/mL was defined as the clearance of HBsAg. Samples with a serum HBsAg titer > 250 IU/mL were diluted to 1:20 and 1:500 with the Architect HBsAg diluent and retested to expand the upper limit of the dynamic range from 250 to 125,000 IU/mL. HBV DNA levels were quantified using the Cobas Taqman assay (Roche Diagnostics, Basel, Switzerland), which has a lower limit of quantification of 12 IU/mL and a linear range of upper detection limit of 1.3 × 10^8^ IU/mL.

### 2.3 Statistical analysis

The study population was randomly allocated into derivation and validation cohorts in a 1:1 ratio. The derivation cohort was used to generate the risk estimation model, and the validation cohort was used to test the final model.

The differences among the categorical or continuous variables were estimated by chi-squared test or *t* test, respectively. Cox proportional hazards model was used to determine the relationship between the clinical variables and the development of HBsAg seroclearance. Simple risk scores were devised by using significant variables obtained from the fully adjusted multivariable model, with *P* < .05. Each score was the weighted sum of those variables for which the weights were rounded to the nearest integer of the corresponding hazard ratio from a Cox regression analysis divided by the smallest hazard ratio. The discrimination capabilities of the risk scores for the development of HBsAg seroclearance were presented in terms of receiver operating characteristic (ROC) curves. The accuracy was measured by the area under ROC curve (AUROC), and the sensitivity, specificity, positive predictive value, and negative predictive value were summarized. The risk scores were then categorized into 2 groups, namely, the low-risk group and the high-risk group, with different cut-off values. The cumulative incidence rates of HBsAg seroclearance according to low-risk and high-risk status were determined using the Kaplan–Meier method. A comparison of the incidence of HBsAg seroclearance according to low-risk and high-risk status was conducted with univariate Cox regression analyses. The significance value of *P* was set at .05. All analyses were performed using Stata version 11.0.

## 3 Results

### 3.1 Characteristics of all patients

Table 1 shows the characteristics of the 201 patients. During the median of 4.4 years of follow-up, 8 patients died and 29 patients were lost to follow-up. Fifty-three patients (26.4%) exhibited serum HBsAg seroclearance, and among these patients, 15 (28.3%) developed anti-HBsAg during follow-up, while 12 (22.6%) developed alanine transaminase (ALT) flare > 80 IU/L before HBsAg seroclearance during follow-up. The cumulative incidence of HBsAg seroclearance was 0% in 1 year, 1.67% in 2 years, 4.03% in 3 years, 7.43% in 4 years, 9.8% in 5 years, 15.24% in 6 years, 17.53% in 7 years, 27.26% in 8 years, 33.5% in 9 years, and 47.07% in 10 years.

**Table 1:**
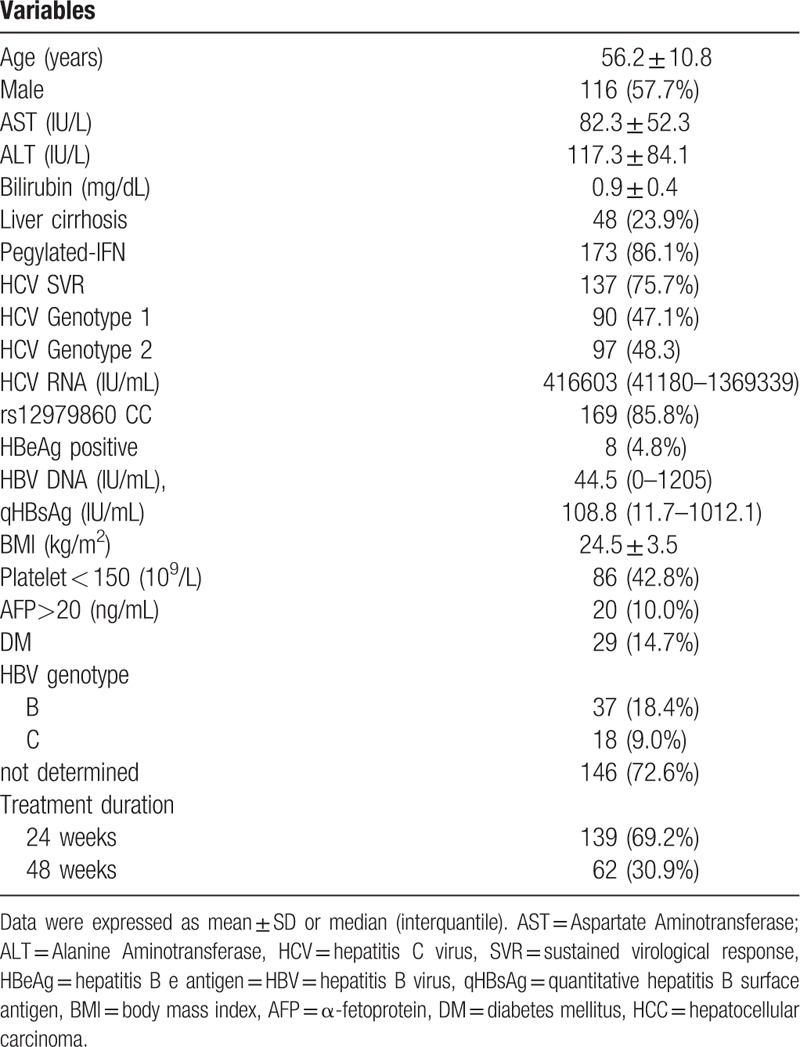
Baseline characterisitic of all patients.

There was no difference in efficacy between the 2 different IFN therapies. The HCV SVR rate was 65.4% in the patients who underwent conventional IFN and RBV therapy, and it was 77.4% in the patients who underwent PEG-IFN and RBV therapy (chi-squared test *P* = .19).

### 3.2 Predictors of HBsAg seroclearance in all patients

Multivariate analysis by Cox regression analysis revealed that HBsAg seroclearance was associated with the following factors: male gender (HR: 3.13, 95% CI: 1.43–6.85, *P* = .004), age > 60 years (HR: 3.30, 95% CI: 1.64–6.66, *P* = .001), and qHBsAg ≤ 100 IU/mL (HR: 6.28, 95% CI: 2.26–17.43, *P* < .001) (Table 2).

**Table 2:**
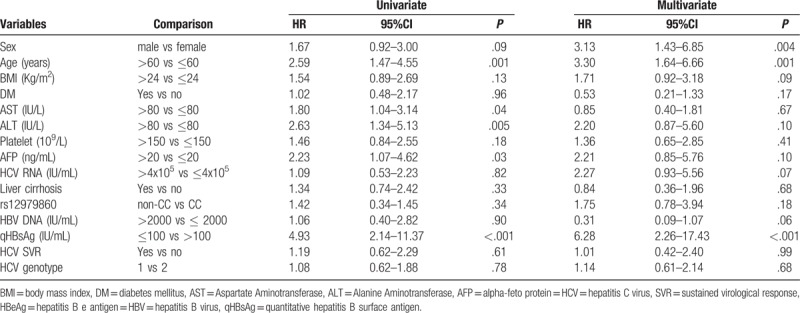
Univariate and multivariate analysis of baseline factors associated with hepatitis B surface antigen seroclearance after antiviral therapy. (All patients).

### 3.3 Characteristics of patients in the derivation and validation cohorts

Table 3 shows the characteristics of the derivation and validation cohorts. There were 100 and 101 cases in the deviation and validation cohorts, respectively. No significant differences existed between the 2 cohorts with regard to their baseline data.

**Table 3:**
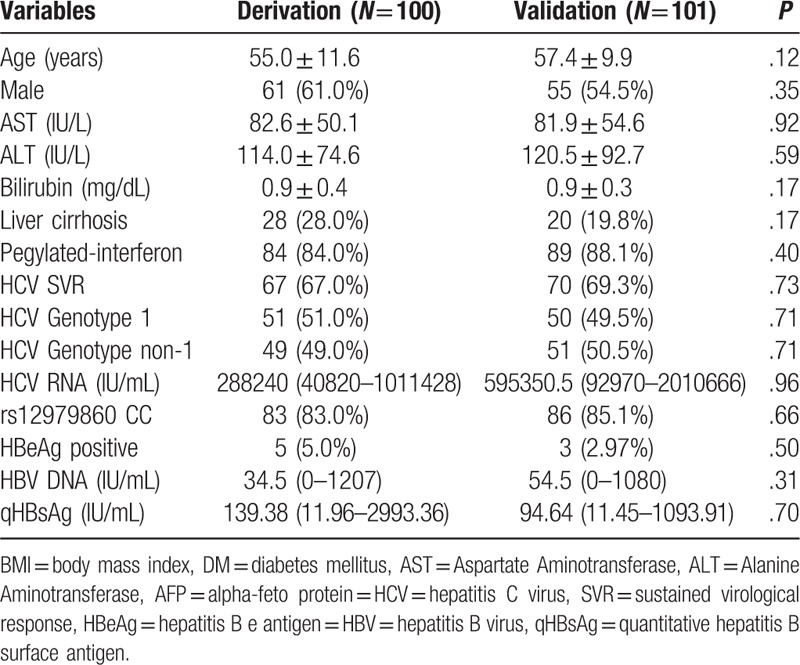
Baseline characteristics of patients in the derivation and validation cohorts.

### 3.4 Predictors of HBsAg seroclearance in the derivation cohort and derivation of prediction score

Multivariate analysis by Cox regression analysis revealed that HBsAg seroclearance was associated with age > 60 years (HR: 5.55, 95% CI: 1.68–18.37, *P* = .005), male gender (HR: 3.88, 95% CI: 1.18–12.80, *P* = .03), and qHBsAg level ≤ 100 IU/mL (HR: 4.87, 95% CI: 1.20–19.74, *P* = .03) (Table 4).

**Table 4:**
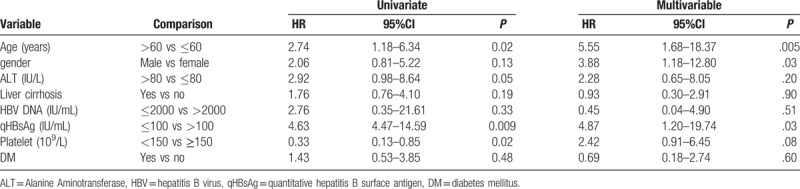
Univariate and multivariate analysis of baseline factors associated with hepatitis B surface antigen seroclearance after antiviral therapy in the derivation cohort.

Subsequently, a simple risk score was devised by using significant variables in the multivariable model according to their contributions of regression coefficients (Table 5). The score ranged from 0 to 3. An ROC curve was calculated, and the AUROC was 71.8%.

**Table 5:**
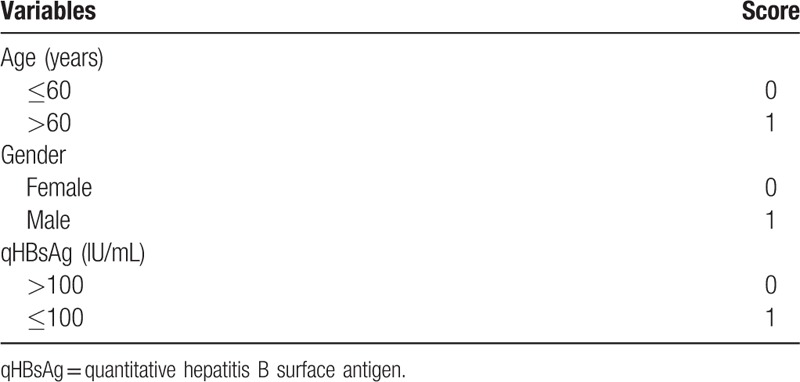
Components of the scoring system to predict hepatitis B surface antigen seroclearance.

For clinical and informative application, the patients were further categorized into two risk groups, the low-risk group (score 0–1) and the high-risk group (score 2–3), which consisted of 66 (66.0%) and 34 patients (34.0%), respectively. In the low-risk and high-risk groups, 8 (12.1%) and 15 patients (44.1%), respectively, developed HBsAg seroclearance during the follow-up period.

**Figure 1:**
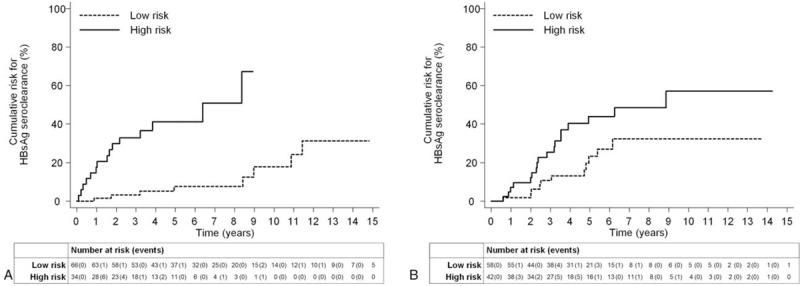
The cumulative incidence of hepatitis B surface antigen seroclearance in patients with low-risk and high-risk category. The 5- year HBsAg seroclearance incidence rates 7.6% in the low-risk category; and 41.1% in the high-risk category in the derivation cohort (HR = 9.01; 95% CI: 3.16–25.70, *P* < .001, Fig. 1A). The 5- year HBsAg seroclearance incidence were 23.4% in the low-risk category, and 43.8% in the high-risk category in the validation cohort (HR = 2.21; 95% CI, 1.04–4.68, *P* = .04, Fig. 1B).

By applying the cut-off point of 2, 15 patients with HBsAg seroclearance were correctly identified and 58 without HBsAg seroclearance were correctly identified. Thus, the sensitivity and specificity of this cut-off value for the detection of HBsAg seroclearance were 65.22% and 75.32%, respectively. The positive and negative predictive values were 44.12% and 87.88%, respectively. The 5-year HBsAg seroclearance incidence rates were 7.6% in the low-risk category and 41.1% in the high-risk category (HR = 9.01; 95% CI: 3.16–25.70, *P* < .001, Fig. 1A).

### 3.5 Validation of results

At the end of follow-up, 30 of 101 patients (29.7%) in the validation cohort developed HBsAg seroclearance. Fifty-eight (57.4%) patients were in the low-risk group, and 43 (42.6%) patients were in the high-risk group. In the low-risk and high-risk groups, 11 (19.0%) and 19 patients (44.2%), respectively, developed HBsAg seroclearance during the follow-up period. The 5-year HBsAg seroclearance incidence rates were 23.4% in the low-risk category and 43.8% in the high-risk category (HR = 2.21; 95% CI, 1.04–4.68, *P* = .04, Fig. 1B).

## 4 Discussion

Patients with a risk score of 0 to 1 had a distinctly different probability of HBsAg seroclearance from those with a prediction score of 2 to 3. Notably, patients with a score 0 to 1 had a “low probability” of HBsAg seroclearance. In the derivation and validation cohorts, 66.0% and 57.4% of the patients, respectively, belonged to the low-risk category and had a low incidence of HBsAg seroclearance during the period of follow-up (12.1% and 19.0%, respectively). In contrast, 34.0% of the derivation cohort patients and 42.6% of the validation cohort patients had a high risk of HBsAg seroclearance, and 44.1% of the derivation cohort patients and 44.2% of the validation cohort patients had HBsAg seroclearance during the follow-up period. The identification of patients’ risk of HBsAg seroclearance could thus help us to determine who would be likely to have a favorable outcome.

There is an increased awareness of HBV reactivation in HBV-HCV coinfected patients treated with DAAs. A recent systematic review and meta-analysis reported that HBV reactivation occurs earlier and is clinically more significant in HBV-HCV coinfected patients treated with DAAs compared to IFN-based therapy. Furthermore, in coinfected patients treated with DAAs, HBsAg seroclearance was not reported.^[20]^ Therefore, PEG-IFN and RBV therapy has the advantage, in comparison with DAAs, of treating both viruses in coinfected patients.

Previous studies showed that 25.6% to 30% of HBV-HCV coinfected patients treated with IFN and RBV dual therapy achieved HBsAg seroclearance during a 5-year follow-up period.^[12,13]^ Factors related to HBsAg seroclearance in HBV-HCV coinfection include liver cirrhosis and HBV DNA negativity at 1 year after the end of treatment,^[13]^ low baseline HBV DNA,^[12,14]^ low baseline HBsAg levels,^[10,15]^ and age ≥ 50 years.^[14]^

In this study, male gender, age > 60 years, and baseline qHBsAg ≤ 100 IU/mL were the independent factors associated with HBsAg seroclearance. These results were generally compatible with those of previous studies.^[10,14,15]^ However, those previous studies^[10,14,15]^ did not show that male gender was associated with HBsAg seroclearance, whereas male gender was associated with HBsAg seroclearance in this study. This discrepancy between those previous studies and this one may be due to the different characteristics of the patients in the various studies and to different covariates being put into the multivariate analyses.

Among 53 patients who exhibited serum HBsAg seroclearance, 15 (28.3%) developed anti-HBsAg during follow-up. A previous study reported that anti-HBs developed in 15 (37.5%) of 40 HBV-HCV coinfection patients with HBsAg seroclearance after IFN and RBV therapy.^[12]^ However, the total numbers of cases in that previous study and our study were small. It is thus difficult to conclude that the rate of seroconversion to anti-HBs was lower is our study than in the previous study.

For patients with HBV-HCV co-infection, the host's immune response is coordinated with each viral replication, usually leading to a predominance of HCV virus.^[21–24]^ Most HBV-HCV coinfected patients have lower serum HBV DNA levels than HBV monoinfected patients.^[25]^ The baseline HBV DNA level [median (interquantile range)] was 44.5 (0–1205) IU/mL in our study. A previous study reported that the baseline HBV DNA level [(median, range)] was 0 (0–1.02 x10^6^) IU/mL in a cohort of patients with HBV-HCV co-infection,^[12]^, a result which is compatible with that of our study.

Low HBsAg and low HBV DNA levels are associated with spontaneous HBsAg seroclearance in HBV monoinfected patients.^[26–28]^ Low HBsAg and low HBV DNA levels are also associated with HBsAg seroclearance in HBV-HCV coinfected patients after IFN and RBV treatment.^[10,12,14,15]^ It is thus interesting to explore whether the HBsAg seroclearance rate after IFN and RBV treatment in HBV-HCV coinfected patients is similar to the rate of spontaneous HBsAg seroclearance in HBV monoinfected patients with low HBsAg and low HBV DNA levels.

In our study, among the 69 patients with HBsAg < 100 IU/mL before therapy, HBsAg seroclearance was noted in 27 patients during the follow-up period, and the incidence rate was 82.49 per 1000 person-years. Of the 106 patients with HBV DNA < 2000 IU/mL before therapy, HBsAg seroclearance was noted in 22 patients during the follow-up period, and the incidence rate was 44.05 per 1000 person-years.

A previous study included 2491 HBV monoinfected patients who were treatment naïve, genotype B or C, HBeAg negative, and cirrhosis-free at study entry. Among the 759 cases with HBsAg < 100 IU/mL at study entry, 363 cases of HBsAg seroclearance occurred during the follow-up period, yielding an incidence rate of 71.43 per 1000 person-years. Among the 719 cases with undetectable HBV DNA (the HBV DNA detection limit was 57 IU/mL), 295 cases of HBsAg seroclearance occurred during the follow-up period, yielding an incidence rate of 57.22 per 1000 person-years. Among 935 cases with HBV DNA detectable -1999 IU/mL, 159 cases of HBsAg seroclearance occurred during the follow-up period, yielding an incidence rate of 20.05 per 1000 person-years.^[28]^

We found that there was no significant difference in the HBsAg seroclearance rate among the patients with HBsAg < 100 IU/mL in our study (82.49 per 1000 person-years, 27 cases within 327.31 person-years) compared with the patients with HBsAg < 100 IU/mL in the aforementioned previous study (71.43 per 1000 person-years, 363 cases within 5081.9 person-years) (Fisher exact test, *P* = .26). There was also no significant difference in the HBsAg seroclearance rate among the patients with HBV DNA < 2000 IU/mL in our study (44.05 per 1000 person-years, 22 cases within 499.43 person-years) compared with the patients with HBV DNA < 2000 IU/mL in the aforementioned previous study (34.69 per 1000 person-years, 454 cases within 13,086.24 person-years) (Fisher exact test, *P* = .16).^[28]^

Little is known about the performance of non-invasive tests for the evaluation of liver fibrosis in HBV-HCV coinfected patients. According to the EASL guideline recommendation, transient elastography can be considered the non-invasive standard for the measurement of liver fibrosis.^[29]^ Only 93 patients in our study underwent liver biopsy, and among them, 30 patients had liver cirrhosis (modified Knodell fibrosis score = 4).^[30]^ None of the 93 patients in our study underwent transient elastography before IFN and RBV treatment. Ten of the 93 patients underwent an endoscopy exam within 6 months before treatment, and none of them had esophageal or gastric varices. All 93 patients underwent an ultrasound exam within 6 months before treatment, and none of them had portosystemic collateral vessels detected by ultrasound. In our study, liver cirrhosis was diagnosed by histology in those who underwent liver biopsy. For those who did not undergo liver biopsy, liver cirrhosis was diagnosed by ultrasound, computerized tomography scan, or magnetic resonance imaging showing cirrhosis combined with portosystemic collateral vessels, or by endoscopy showing esophageal or gastric varices.^[19]^ Although portosystemic collateral vessels are a 100% specific (pathognomonic) sign of portal hypertension, such that liver cirrhosis can be diagnosed without liver biopsy with this sign,^[19]^ among the 30 patients with biopsy-proven liver cirrhosis in our study, none of them had portosystemic collateral vessels detected by ultrasound. As such, it can be concluded that liver cirrhosis might be underdiagnosed by using this non-invasive criterion in our study. According to the American Association for the Study of Liver Diseases (AASLD) guideline recommendation, patients with compensated cirrhosis should be substage into those with mild portal hypertension and those with clinically significant portal hypertension (CSPH). The presence of portosystemic collaterals on imaging or gastroesophageal varices on endoscopy is sufficient to diagnose CSPH.^[19]^ In conclusion, using this non-invasive criteria to diagnose liver cirrhosis in our study could have led to the underdiagnosis of liver cirrhosis in those without CSPH.

Our study had several limitations. First, it was a retrospective study. Second, the number of cases included in the study was small. However, it is difficult to conduct a prospective study to enroll more coinfected patients treated with IFN-based therapy in the era of DAAs. Third, using the non-invasive criterion used in this study to diagnose liver cirrhosis could have caused liver cirrhosis to be underdiagnosed in those without CSPH. The strength of our study, meanwhile, was that by using a simple scoring system, we could predict HBsAg seroclearance in chronic hepatitis B and C coinfected patients treated with IFN and RBV.

In conclusion, we developed a simple scoring system to predict HBsAg seroclearance in chronic hepatitis B and C coinfected patients treated with IFN and RBV. This predictive scoring system is simple and could be clinically applicable. The benefit of IFN-based therapy in coinfected patients, as shown in a previous study, is that it decreases the risks of developing HCC, liver-related mortality, and all-cause mortality compared with a lack of treatment.^[16]^ However, there is no relevant data regarding the use of DAAs in coinfected patients currently. A future study to investigate whether therapy with DAAs decreases the risks of HCC, liver-related mortality, and all-cause mortality in HBV-HCV coinfected patients is thus needed.

## Funding/Support Statement and Acknowledgment

This study was supported by Grant CMRPG8G1181 from the Chang Gung Memorial Hospital-Kaohsiung Medical Center, Taiwan. The funders had no role in the study design, data collection and analysis, decision to publish, or preparation of the manuscript.

## Author contributions

**Conceptualization:** Yi-Hao Yen.

**Data curation:** Yi-Hao Yen.

**Formal analysis:** Yi-Hao Yen.

**Funding acquisition:** Yi-Hao Yen.

**Investigation:** Yi-Hao Yen.

**Methodology:** Yi-Hao Yen.

**Project administration:** Yi-Hao Yen.

**Resources:** Yi-Hao Yen.

**Software:** Yi-Hao Yen.

**Supervision:** Kwong-Ming Kee, Fang-Ying Kuo, Kuo-Chin Chang, and Tsung-Hui Hu, Sheng-Nan Lu, Jing-Houng Wang, Chao-Hung Hung, and Chien-Hung Chen.

**Validation:** Yi-Hao Yen.

**Visualization:** Yi-Hao Yen.

**Writing – original draft:** Yi-Hao Yen.

**Writing – review & editing:** Yi-Hao Yen.

Tsung-Hui Hu orcid: 0000-0002-9172-1967.
